# Q-Switch Nd:YAG Laser-Assisted Elimination of Multi-Species Biofilm on Titanium Surfaces

**DOI:** 10.3390/ma13071573

**Published:** 2020-03-29

**Authors:** Melanie Namour, Tim Verspecht, Marwan El Mobadder, Wim Teughels, Andre Peremans, Samir Nammour, Eric Rompen

**Affiliations:** 1 Department of Dental Sciences, Faculty of Medicine, University of Liege, 4000 Liege, Belgium; melanienamour@gmail.com (M.N.); marwan.mobader@gmail.com (M.E.M.); erompen@hotmail.be (E.R.); 2 Department of Oral Health Sciences, University of Leuven (KU Leuven), Kapucijnenvoer 33, 3000 Leuven, Belgium; tim.verspecht@kuleuven.be; 3 Department of Oral Health Sciences, University of Leuven (KU Leuven) and Dentistry, University Hospitals Leuven, Kapucijnenvoer 33, 3000 Leuven, Belgium; wim.teughels@kuleuven.be; 4 Laboratoire Physique de la Matière et du Rayonnement (P.M.R.), Université de Namur, 5000 Namur, Belgium; Andre.peremans@gmail.com

**Keywords:** biofilm, biofilm removal, dental implant, laser, periimplantitis, titanium surface

## Abstract

(1) Background: The relatively high prevalence of peri-implantitis (PI) and the lack of a standard method for decontamination of the dental implant surface have pushed us to conduct further research in the field. Bacterial biofilms were found to play a primordial role in the etiology of PI. Therefore, the aim is to evaluate the efficacy of a laser-assisted elimination of biofilm protocol in the removal of a multi-species biofilm on titanium surfaces. (2) Methods: In total, 52 titanium discs (grade 4) were used. The study group consisted of 13 titanium disks contaminated with multi-species biofilms and subsequently irradiated with the laser (T + BF + L). The control groups consisted of the following types of titanium disks: 13 contaminated with multi-species biofilms (T + BF), 13 sterile and irradiated (T + L), 13 sterile and untreated (T). Q-Switch Nd:YAG laser Irradiation parameters were the following: energy density equal to 0.597 J/cm^2^ per pulse, power equal to 270 milliwatt per pulse, 2.4 mm of spot diameter, and 10 Hz repetition rate for pulse duration of six nanoseconds (ns). The laser irradiation was made during 2 s of total time in non-contact and at 0.5 mm away from the titanium disc surface. After treatment, presence of biofilms on the disks was evaluated by staining with crystal violet (CV), which was measured as optical density at six hundred thirty nm, and statistical analyses were done. (3) Results: the optical density values were 0.004 ± 0.004 for the study group T + BF + L, 0.120 ± 0.039 for group T + BF, 0.006 ± 0.003 for group T + L, and 0.007 ± 0.007 for group T. For the study group, laser treatment resulted in a total elimination of the biofilm, with mean values statistically significantly lower than those of contaminated titanium surfaces and similar to those of sterile titanium surfaces. (4) Conclusions: Our irradiation protocol provided a significant elimination of the multi-species biofilm on titanium surfaces. Laser treated titanium surfaces were biofilm-free, similar to the sterile ones.

## 1. Introduction

The common treatment after tooth loss is the placement of a dental implant [1]. The survival of the placed dental implant depends on several criteria such as the absence of mobility, the lack of a radiolucent image in the peri-implant tissue, a bone loss of almost 200 μm each year following the first 12 months of placement, the absence of any chronic and irreversible signs and the absence of any symptoms [2]. Unfortunately, the placement of the endogenous dental implant results in several complications associated with an inflammation that surrounds the periodontal tissue surrounding the implant and other pathological conditions [3,4]. Peri-implantitis (PI) is defined as the inflammation of the soft tissue and the loss of bone beyond the biological remodeling process around the implant [3,4,5]. Even more so, Lindhe and Meyle established that incidence of PI ranges between 28% and 56% [6]. 

Many studies have been focusing on the elimination of the biofilm adherent to the implant surface, which will result in a recession of the inflammation around the implant. This elimination, cleaning or decontamination of the dental implants is hard to obtain in the micro and nano treated surfaces. In fact, the roughness of the surface will improve the osseointegration but will facilitate the retention of the biofilm and will result in a compromised ability to clean the surfaces [4].

Literature indicates that biofilms on the surface of the dental implant presents a primordial factor in the progression and appearance of peri-implantitis, which is currently considered as a poly-microbial anaerobic infection characterized by a large spectrum of periodontopathic and non-periodontopathic bacteria [4,7]. For this reason, the elimination of these biofilms is still considered as the treatment of choice for PI [7,8].

In the last decades, more knowledge has been gained about the pathophysiology of PI and the tight relation between PI and the pathogenic multispecies biofilm that is found in the peri-implant tissue [9,10]. Furthermore, activator nuclear factor kappa-B, osteoprotegerin, and sclerostin levels were found in a study to be implicated in the appearance or progression of PI, suggesting their possible role as candidates for prognostic biomarkers in the diagnosis of the PI [11]. Isola et al., revealed the association between periodontitis in ischemic heart disease patients and the low serum and salivary vitamin C and antioxidant levels [12]. Whereas, a study showed that the Cyclophilin A may be an early signal for peri-implantitis [13]. In addition, the fibroblasts may be implicated in the PI pathogenesis by increasing the response to matrix breakdown and vascularity, consequently stimulating the promotion migration and preservation of infiltrates into the site [14].

Despite the current progress in understanding PI, there is no gold standard in the management of PI. It appears that the proposed conventional treatments do not lead to complete debridement of the implant surfaces exposed to bacterial contamination without destroying the original implant surface roughness and its physical properties [15]. For this reason, innovative approaches are being suggested in order to assist the mechanical debridement, as well as enhancing the decontamination for the sake of having a re-osseointegration [16,17,18].

Among the numerous treatment protocols that are being proposed for the management of PI, the use of lasers could be considered a promising technique [16,18]. This is because of its cleaning effect, i.e., the microbial destruction it causes, and the consequent biological effects might have a significant impact resolution process of PI [19,20].

An in vitro study showed that when irradiation with short pulse duration (in ns) with Q-switch Nd:YAG laser was used the treatment has led to a significantly clean implant surfaces, without generating a dramatic increase of implant surface temperature [19]. Specifically, only 1 °C was seen, which is considered as acceptable for the surrounding periodontal tissue (below 10 °C) [19]. In this study, Namour et al. showed that the irradiation of the contaminated implant surfaces resulted in a carbon mass percentage reduction similar to that obtained for sterile titanium implant surfaces [19]. These findings mean that the treatment is able to completely clean the previously contaminated implants without any damage to the surrounding periodontal tissue and with a percentage of carbon mass statistically significantly equal to a sterile implant surface [19]. However, the study did not focus on the analysis of the biofilm elimination from contaminated surfaces [19]. 

In this study, the capability of the Q-switch Nd:YAG laser irradiation to eliminate multi-species biofilms grown on titanium surfaces was evaluated. The laser irradiation parameters were the same used by Namour et al. [19], to ensure the safety and effectiveness of the treatment. 

Hence, the objective is to assess the efficiency of our proposed protocol with the Q-switch Nd:YAG laser in the removal of multi-species biofilm grown on titanium surfaces. The null hypothesis is that the treatment would not lead to a significant eradication of the biofilms.

## 2. Material and Methods

### 2.1. Design of the Study

**Figure 1:**
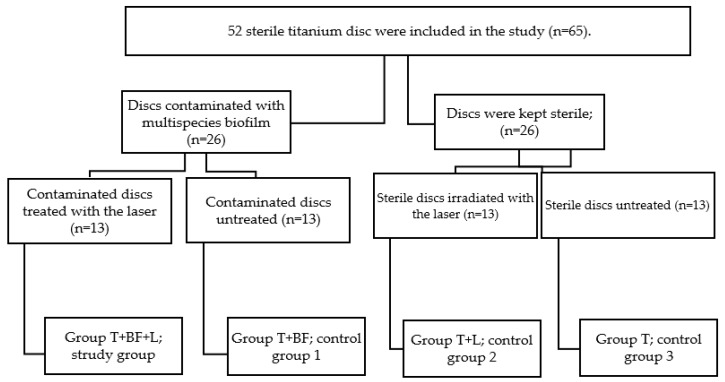
Study design, experimental and control groups in the study. T—titanium discs; BF—biofilm; L—laser treatment.

In total, fifty-two titanium discs (Straumann dental implant Switzerland, Straumann AG, Basel) were used in this study. The discs are titanium grade 4, sandblasted and acid-etched treated titanium. The size of each disc was 5 mm in diameter and 1 mm in thickness. Twenty-six titanium disks were contaminated with a multi-species biofilm (*n* = 26) and twenty-six were kept sterile (*n* = 26). Among the 26 contaminated titanium discs, 13 were irradiated and were considered as the study group (group T + BF + L; *n =* 13) and 13 were left untreated and were considered as the first control group (T + BF; *n* = 13). From the 26 sterile discs, 13 were irradiated by the laser and were considered as a second control group (T + L; *n* = 13). Thirteen were kept sterile without any irradiation and were considered as the third control group (group T; *n* = 13) (Figure 1). The Ethical committee of the University of Liège, Belgium, decided that this study does not require prior ethical committee approval.

### 2.2. Bacterial Strains, Media, and Growth Conditions

Fourteen bacterial strains were used to form multispecies biofilms. The following bacterial strains were included: the representative pathogens are *Streptococcus sobrinus* ATCC 33478, *Aggregatibacter actinomycetemcomitans* ATCC43718, *Fusobacterium nucleatum* ATCC10953, *Prevotella intermedia* ATCC 25611, *Porphyromonas gingivalis* ATCC 33277, *Streptococcus mutans* ATCC 25175. *Actinomyces naeslundii* ATCC 51655, *Actinomyces viscosus* ATCC 15987, *Streptococcus cristatus* ATCC 49999, *Streptococcus gordonii* ATCC 49818, *Streptococcus mitis* ATCC 49456, *Streptococcus oralis* DSM 20627, *Streptococcus sanguinis* LMG 14657, *Streptococcus parasanguinis* DSM 6778, as well as *Veillonella parvula* DSM 2008 were included as beneficial or commensal bacteria. 

All species were grown on blood agar (Oxoid, Ltd., Basingstoke, UK) added by means of five µg/mL hemin, one µg/mL menadione (Sigma-Aldrich Co., St.-Louis, MO, USA) in addition to five percent sterile horse blood (E&O Laboratories Ltd, Bonnybridge, Scotland). *A. actinomycetemcomitans*, *S. cristatus*, *S. mitis, S. gordonii*, *S. oralis, S. mutans*, *S. parasanguinis*, *S. salivarius*, *S. sobrinus,* and *S. sanguinis* were grown at 37 °C in a 5% CO_2_ environment. *Actinomyces naeslundii*, *Actinomyces viscosus*, *F. nucleatum*, *P. gingivalis*, *P. intermedia,* and *V. parvula* were grown at thirty-seven degree Celsius for anaerobic conditions (80% diazote, ten percent dihydrogen, and 10% CO_2_). 

Single species planktonic cultures were prepared by collecting bacteria from the blood agar plates than inoculating them in ten milliliter brain heart infusion broth (BHI) (Difco Laboratories, Detroit, MI, USA) followed by incubation under identical conditions as the blood agar plates, depending on bacterial species. The optical densities were assessed at 600 nm through spectrophotometry (OD600; Gene Quant Spectrophotometer, Biochrom Ltd., Cambridge, UK). 

Multi-species biofilms were grown in modified BHI broth, consisting of 37 g/L BHI added with 2.5 g/L mucin from porcine stomach type-III (Sigma-Aldrich Co, St.-Louis, USA), 1.0 g/L yeast extract (Oxoid, Basingstoke, UK), 0.13 gram per liter cysteine HCl (Merck-Calbiochem, San Diego, USA), 2.0 g/L sodium bicarbonate (Merck, Darmstadt, Germany) and 3.65 g/L 0.25% glutamic acid (Merck-Calbiochem, San Diego, USA) Multi-species biofilms were grown at thirty-seven degree Celsius with microaerophilic circumstances (six percent O_2_, seven percent CO_2_, seven percent H_2_, and eighty-percent N_2_).

#### Bioreactor-Derived Multi-Species Community and Multi-Species Biofilms

The multi-species community ^®^ B- Benchtop Bioreactor Controller, Sartorius BioTech GmbH, Goettingen, Germany) with a culture vessel containing 750 mL modified BHI broth supplemented with five mg milligram per liter hemin, one milligram per liter menadione and 200 microliter per liter Antifoam Y-30 (Sigma-Aldrich Co., St.-Louis, MO, USA). The medium was continuously stirred (300 rpm) at a constant temperature (37 °C), bubbled with 100% N_2_ and 5% CO_2_, and pH set at 6.7 ± 0.1. After 24 h to allow the medium to equilibrate, an ON culture of *S. mitis* was inoculated into the vessel and grown until late exponential stage; after which remaining bacterial ON cultures were adjusted to an OD600 ~ 1.2–1.4 and added to the vessel. A stable 14-species community was allowed to establish during 48 h and was afterwards kept in continuous culture with exchange of 200 mL medium every 24 h [21]. A more detailed protocol and parameter settings can be found in a study of Slomka et al. [21].

Multi-species biofilms were grown horizontally on titanium discs on the bottom of a 48-well plate. Samples from the bioreactor-derived multi-species community underwent one: ten dilution in fresh adapted brain heart infusion medium, after which 500 µL was added to each well containing a disc. Biofilms were allowed to establish during 48 h in microaerophilic conditions (six percent O_2_, seven percent CO_2_, seven percent H_2_, and eight percent N_2_) at 37 °C and 170 rpm [21].

Discs containing the established biofilms were dip-rinsed in phosphate buffered saline with a 7.4 pH to eliminate all the cells that are unattached. Afterwards, discs were transferred to a new 48-well plate and subsequently subjected to the laser treatment.

### 2.3. Treatment Protocol and Laser Irradiation

The Q-switch Nd:YAG laser (1064 nm wavelength, Q-smart 850, Lumibird, Lannion city, France) was used for the treatment of the titanium discs. A laser power meter (Ophir Spiricon Europe GmbH, Darmstadt, Germany) was used to make sure that the irradiation conditions are successfully transported at the target. Irradiation settings delivered per pulse on the target (surface of the titanium discs) were the following: a 0.597 J/cm^2^ energy density per pulse, 270 mW Power per pulse, the diameter of the spot of the laser was equal to 2.4 mm; the repetition rate was 10 Hertz (Hz) for a pulse duration of six nanoseconds. The irradiation was made in a non-contact mode with a total of two seconds and a distance of Irradiation was performed during a total time of 2 s in a non-contact mode at a distance of 500 μm from the surface of the titanium discs. The selection of the energy was obtained from the theoretical modeling of the laser fluency at the level of damaged threshold of the titanium made in a previous study [19]. Directly after the laser irradiation, the concerned titanium disc was stocked in a sterile box for analysis.

### 2.4. Biofilm Staining with Crystal Violet (CV) 

Following laser treatment, the presence of multi-species biofilms on the discs was evaluated by staining them with crystal violet (CV). The discs were dip-rinsed twice in 445 µL PBS (pH 7.4), subsequently transferred to an empty well in a new 48-well plate and briefly left to dry. Next, biofilms were fixated for 20 min in 445 µL 96% ethanol. Disks were again transferred to a new, empty well and left to dry. Biofilms were stained by adding 445 µL of CV (1%) for 15 min, after which excess/non-bound CV was removed by repeatedly (average of eight times) dip-rinsing the disks in distilled water and disks were allowed to dry. Finally, bound CV dye was solubilized by with 334-microliter acetic acid (5%) to each well during 45 min. The optical density of the resulting solution was evaluated at six hundred thirty nanometer (OD_630 nm_).

### 2.5. Statistical and Data Analysis

Prism 5^®^ software (GraphPad Prism 5, Graph Pad Software, Inc., San Diego, CA, USA) was used to perform statistical analysis. Statistical significance was considered when *p* was less than 0.05. Confidence level of the study was proposed to be ninety-nine percent (99%) with *p* < 0.001, which is considered to have a very high significance. In addition, Mean and Standard deviations were calculated. Kolmogorov and Smirnov test was used for the Normality tests. Two-way Paired ANOVA coupled with a Newman–Keuls Multiple comparison test (post hoc test) were used. 

## 3. Results

**Figure 2:**
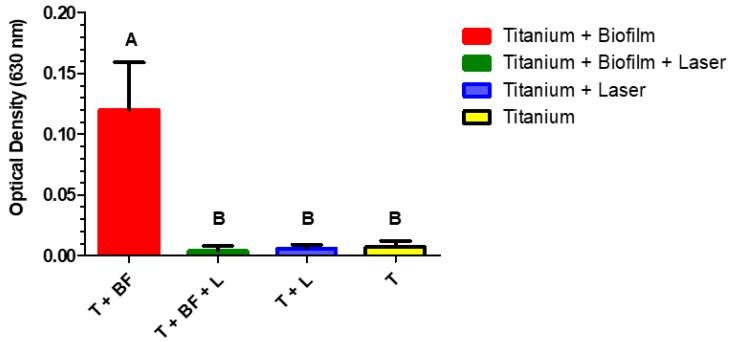
Amounts of biofilm present on titanium discs in the different groups as measured through crystal violet staining. Values in all groups passed the Normality tests (Kolmogorov and Smirnov test). Identical letters indicate the absence of a statistically significant variance, while difference in letters indicate a statistically significant difference. *p*-value < 0.0001.

The amount of biofilm present on the discs in the different groups was measured through CV staining then expressed by means of the mean (± standard deviation) values of the OD_630 nm_, which reflects the crystal violet’s absorbance. For the T + BF + L group (study group), the OD_630 nm_ was 0.042 ± 0.004, for the T+BF group (control group 1) 0.158 ± 0.039, for the T+L group (control group 2) 0.044 ± 0.004, and for the T group (control group 3) 0.053 ± 0.007. All values in all groups passed the normality tests. The OD_630 nm_ value obtained for the study group T + BF + L was statistically significantly lower than for the T + BF group. In addition, the OD_630 nm_ value obtained for the study group T + BF + L was similar to that obtained for the different control groups with sterile discs (Group T and Group T + L), as reflected by the absence of a difference between the study group and the control groups (the difference in values was not statistically significant). Therefore, laser treatment resulted in the complete elimination of the biofilms from the titanium discs, with statistically significantly lower OD_630 nm_ values than for untreated, contaminated titanium surfaces and similar to those observed for sterile titanium surfaces (Figure 2). The difference was not statistically significant when the amounts of biofilm in the group T + BF + L was compared to the group T. Therefore, the null hypothesis was rejected.

## 4. Discussion

The present study showed that the suggested treatment with the Q switch Neodymium-Doped Yttrium-Aluminum Garnet laser was able to remove efficiently the multispecies biofilm on the surfaces of the titanium discs. After treatments, there was no statistical difference in the amount of biofilm present on the sterile titanium discs when compared to the once contaminated and then laser-treated. In fact, the amount of biofilms on the contaminated titanium discs was similar to those of the sterile ones. 

The safety of the present protocol was confirmed previously by Namour et al. [19]. In fact, authors showed that the irradiation parameters used did not provoke morphological changes on the implant surface nor an increase of temperature greater than 1 °C.

In this study, crystal violet staining was used to quantify the amounts of biofilm present on the titanium surfaces, since it is a simple, quick and reliable method for the indirect quantification of biofilm mass [22]. 

The use of the short duration in nanoseconds with the Q-switch Nd:YAG laser have the ability to induce a temperature increase; which is sufficient to produce desorption of the contaminant substrates without producing a change in the physical properties and the destruction of the original porosities and roughness of implant surface [19]. These short pulses of five nanoseconds have led to a decrease of up to two orders of magnitude of the required laser output power and, thus, a reduction of the rise in temperature during the decontamination process [19]. For this reason, in the current study, we used similar irradiation conditions, which led to a total biofilm and bacterial elimination. Additionally, one of the potential advantages of the Q-switch Nd:YAG laser is the thin flexible light-conductor fiber system that could help the operator to attain almost any desired location and, therefore, practically all areas of the implant can be easily reached and treated [23,24]. 

The thermal effect produced by the light and results in the elimination of an adsorbed material from a metallic surface is defined as the thermal-photo-desorption. A large number of studies have focused on the photo-desorption of organic molecules from a metal surface [25]. The breaking of the adsorbate-substrate bonds requires the absorption of multiple photons [26]. In the infrared spectrum, the breaking of the bond between the adsorbate and the substrate is severe because the photon energy is inferior to the substrate adsorption energy [25,26]. Anyhow, the dominant mechanism of energy uptake from the laser beam is the absorption. Consequently, thermal processes dominate the laser-induced photo desorption process in the infrared range. It is well recognized that the property in the infrared is quite wavelength independent, but temperature transience metal absorption and contaminant desorption are strongly pulse duration dependent. Biological tissue absorption can be in the order of 1 cm^−1^ near 1.064 μm, and 100 cm^−1^ near 2.94 μm [25,26,27]. Hence, it is not possible to settle that hundred microns thick biofilms can present significant absorption in the one percent or sixty-five percent of near 1064 and 2940 nm, respectively [25].

Understanding the above, and according to the results of the study, it can be concluded that following the irradiation with the Q switch Nd:YAG, raise of temperature which resulted in heating the titanium surface was enough to break the chemical bond between the contaminant or the biofilm and the titanium surface. However, the rise in temperature was superficial, no more than 850 nm in depth, not enough to cause any liquefaction or damage of the titanium surface, which is once again, great enough to cause a de-bonding between the titanium surface and the contaminants [19]. Therefore, the biofilm was removed successfully without any side effect on the titanium surface [28,29,30].

Literature from different fields of dentistry has proven that lasers within the infrared range exhibit a significant antibacterial effect and are able to deactivate bacterial toxins [31,32,33]. The irradiation with the laser’s energy results in the denaturation of the protein at a temperature of fifty degree Celsius, a protein coagulation at a temperature of sixty degree Celsius, a vaporization and ablation at a hundred degree Celsius and tissue carbonization when the temperature surpass two hundred degree Celsius. [34,35]. Target chromophores of near-infrared lasers are the pigmentation, which can be found in some types of bacteria. The scattering and deep penetration of the Nd:YAG light and its high photothermal potential has led to its use for decontamination [34,36,37]. In fact, Nd:YAG laser irradiation has resulted in a reduction of interleukin-1β, which has a stimulating effect on the bone resorption [38,39,40].

Numerous studies have been conducted in order to enhance the treatment of PI. Al-Hashedi et al. showed that titanium brushes presents better removal effect compared to curettes and Er:YAG laser [41]. A study on the efficacy of antimicrobial agents in removal of multispecies oral biofilm showed that rinsing titanium surfaces with 0.9% NaCl removes the majority of biofilm but with a persistence of all bacterial species [42]. The study also showed that neither chlorhexidine gel, phosphoric acid gel, cetrimide 0.1%, chlorhexidine, nor acid éthylènediaminetétraacétique (EDTA) were able to have a superior disinfection ability compared to a double saline rinse group [42]. Wiedmer et al. found that the use of hydrogen peroxide titanium dioxide suspension results in a better bactericidal effect compared to the use of hydrogen peroxide alone and can cause a delay of the bacterial regrowth [43]. A study that compared the Erbium-Doped Yttrium Aluminum garnet laser, low-level laser therapy and bur designed for the titanium in addition to citric acid in the management of PI found that the burrs coupled with citric acid had a better improvement the regeneration of the surrounding bone [44]. According to the study, the difference was significant statistically [44]. Furthermore, Isola et al. reported that patients with coronary heart disease and periodontitis plus coronary heart disease shows higher serum and salivary levels of Endothelin 1 compared to patients with periodontitis and those that were healthy. Additionally, according to their study only C-reactive protein remained a major predictor of increased ET-1 concentrations in both serum and saliva [45]. 

Hence, decontamination remains an area of intense research and development in medicine, more trials are needed in order to settle the high efficiency of the Q-Switch Nd:YAG laser to eliminate the biofilm from different titanium surfaces and grades used in dental or medical field. 

## 5. Conclusions

In conclusion, our study showed that our irradiation conditions for Q-Switch Nd:YAG laser-assisted biofilm removal has provided a significant elimination of the biofilm on the titanium surfaces. The treated surfaces showed similar amounts of biofilms when compared to sterile, biofilm-free titanium surfaces.
